# The Effect of Laparoscopic Sleeve Gastrectomy on Body Mass Index and the Resolution of Other Metabolic Syndrome Components in Patients over 50 Years Old during a Two Year Follow-Up

**DOI:** 10.3390/jcm13195662

**Published:** 2024-09-24

**Authors:** Aleksander Łukaszewicz, Paulina Głuszyńska, Zuzanna Razak Hady, Patrycja Pawłuszewicz, Jerzy Łukaszewicz, Hady Razak Hady

**Affiliations:** 1 1st Clinical Department of General and Endocrine Surgery, Medical University of Białystok Clinical Hospital, 15-276 Białystok, Poland; 2 1st Clinical Department of General and Endocrine Surgery, Medical University of Białystok, 15-569 Białystok, Poland

**Keywords:** laparoscopic sleeve gastrectomy, bariatric surgery, metabolic surgery, metabolic syndrome, comorbidities, hypertension, dyslipidemia, type 2 diabetes

## Abstract

**Background/Objectives**: Metabolic syndrome, defined by the coexistence of central obesity, dyslipidemia, hypertension, and insulin resistance, is a significant contributor to increased cardiovascular morbidity and mortality in the aging population. We aimed to determine whether age influences the efficacy of LSG in treating obesity-related comorbidities. **Methods**: A retrospective analysis of lipid profiles, glycemic and clinical parameters was conducted in a group of 786 patients in two age groups (under 50 years old and over 50 years old) who underwent laparoscopic sleeve gastrectomy with follow-ups 1, 3, 6, 12 and 24 months after surgery. **Results**: There was a significant improvement in lipid metabolism with no significant differences between the two age groups in these parameters throughout the observation period. Furthermore, there was significant weight loss (54.82 kg vs. 54.56 kg) and BMI reductions (47.71 kg/m^2^ vs. 47.01 kg/m^2^ to 29.03 kg/m^2^ vs. 30.73 kg/m^2^). Total cholesterol decreased from 198 mg/dL to 184.9 mg/dL (<50 years old) and from 206.4 mg/dL to 193 mg/dL (>50 years old). LDL dropped from 136.2 mg/dL to 116.7 mg/dL and from 141.0 mg/dL to 121.0 mg/dL. Mean HbA1c decreased to comparable levels (5.66% vs. 5.53%). Both groups showed similar rates of remission for type 2 diabetes and hypertension. **Conclusions**: Our findings suggest that LSG is an effective method for treating components of metabolic syndrome regardless of age, supporting its use as a therapeutic tool for older patients.

## 1. Introduction

Obesity, a global epidemic, presents a significant public health challenge in the 21st century, with the estimation that 1 billion people over the world will be obese [1]. Characterized by an excessive accumulation of body fat, obesity is not merely a cosmetic concern but a complex condition with serious social and psychological dimensions. It affects nearly every aspect of health, from reproductive and respiratory function to memory and mood [2,3,4]. The prevalence of obesity has shown a staggering increase over the past decades, leading to a substantial rise in obesity-related health conditions. This trend is particularly alarming among the population aged 50 years and older, since they are already vulnerable to age-related health issues.

Central to the obesity crisis is the development of metabolic syndrome, a cluster of conditions that occur together, increasing the risk of heart disease, stroke and type 2 diabetes [5]. Metabolic syndrome is defined by a combination of factors including increased blood pressure, hyperglycemia, excess body fat around the waist and abnormal cholesterol or triglyceride levels [6]. The pathophysiology of metabolic syndrome is complex and multifactorial, involving genetic, metabolic and environmental factors [7].

The treatment of metabolic syndrome involves a multifaceted approach, targeting its individual components through lifestyle modifications and pharmacological interventions [8,9]. Lifestyle changes such as diet, exercise and weight loss are first-line therapies. Pharmacologic treatments are available for controlling hypertension, hyperglycemia and dyslipidemia, which are core aspects of the syndrome. However, the efficacy and safety of these treatments can vary in older patients due to physiological changes associated with aging and the presence of comorbidities [10].

In the context of obesity, particularly severe obesity, bariatric surgery has emerged as a potent intervention. Among the surgical options, laparoscopic sleeve gastrectomy (LSG) is the most performed one due to its relative simplicity, efficacy, and safety profile [11,12,13]. LSG not only assists in significant weight loss but also in the amelioration of obesity-related comorbidities [14,15,16,17]. LSG exerts significant neurohormonal effects that enhance its efficacy in treating obesity and metabolic syndrome. A marked reduction in ghrelin levels is observed post-LSG, leading to decreased appetite and food intake. Additionally, the surgery increases the secretion of incretin hormones such as GLP-1 and PYY, which improve satiety and glycemic control. Elevated bile acid levels after LSG further enhance glucose homeostasis and insulin sensitivity through the activation of FXR and TGR5 receptors [18,19,20]. Nevertheless, the effectiveness of LSG in resolving metabolic syndrome, especially in individuals over the age of 50, remains an area requiring further exploration. This age group presents unique challenges and considerations, given the physiological changes in aging and the increased likelihood of concurrent medical conditions.

The purpose of this study is to investigate the impact of laparoscopic sleeve gastrectomy on the resolution of metabolic syndrome in patients over 50 years old. This study aims to elucidate the role of LSG in managing the intricate interplay of obesity and metabolic syndrome in this particular demographic, thereby contributing to more effective and tailored therapeutic strategies in the future.

## 2. Materials and Methods

An observational and retrospective study was conducted from January 2010 to January 2023 by a single surgical team. A total of 786 patients who underwent laparoscopic sleeve gastrectomy as a primary procedure were included (Table 1). Follow-up was scheduled 1, 3, 6, 12 and 24 months after the surgery. Tested parameters included fasting glucose, HbA1C, triglycerides, high-density lipoprotein, low-density lipoprotein and total cholesterol levels. All the tests were performed by the same laboratory at the Medical University of Bialystok Clinical Hospital. Additionally, body weight was measured and any changes in the use of antihypertensive or antidiabetic medications were noted.

The procedures were performed at the Medical University of Bialystok Clinical Hospital. Patients were qualified for surgery in accordance with the Polish Guidelines on Metabolic and Bariatric Surgery [21]. The exclusion criteria were as follows: obesity-related endocrine diseases, severe uncontrolled psychiatric illnesses, severe coagulopathy, women planning on pregnancy within two years after a potential surgery, life-threatening diseases in a short time (e.g., recent cardiac arrest, severe chronic obstructive pulmonary disease).

All surgeries were performed by the same team of surgeons in a standardized way, using a 36-Fr diameter bougie with a dissection line beginning approximately 6 cm from the pylorus up to the angle of His, using a 60 mm linear stapler. The leak test was performed with the use of methylene blue solution and air. Patients were discharged on the first postoperative day if no complications occurred. All patients were informed about the risk of surgical treatment and written consent was obtained from every participant before the surgery.

Metabolic syndrome was diagnosed using National Heart, Lung, and Blood Institute, American Heart Association, World Heart Federation, International Atherosclerosis Society, and International Association for the Study of Obesity (AHA/NHLBI + IDF) criteria. It defines MetS as any three of the following: impaired glucose metabolism (FPG ≥ 100 mg/dL or antidiabetic treatment), hypertension (BP ≥ 130/85 or antihypertensive treatment), elevated triglycerides (TG ≥ 150 mg/dL or on treatment), reduced HDL-C (HDL-C < 40 mg/dL in men and <50 mg/dL in women, or on treatment) and abdominal obesity (increased waist circumference with normal values depending on population and country-specific definitions) [6].

Postoperative weight loss was expressed in terms of percent total weight loss (%TWL), percent excess weight loss (%EWL) and percent excess BMI loss (%EBMIL). The following formulas were used:

- Percent total weight loss: %TWL = (initial weight-current weight)/(initial weight) × 100;
- Percent excess BMI loss: %EBMIL = (initial BMI-postoperative BMI)/(initial BMI-25) × 100;
- Percent excess weight loss: %EWL = (initial weight-postoperative weight)/(initial weight-ideal weight) × 100, where ideal weight is defined by the weight corresponding to a BMI of 25 kg/m^2^.

## 3. Data Analysis

The statistical analysis of the results was conducted using GraphPad Prism 9.0.0 software (GraphPad Software, San Diego, CA, USA). The assessment of the normality of distribution was carried out using the Shapiro–Wilk W test, while the assessment of the homogeneity of variances was performed using Levene’s test. For comparisons between two groups, the Mann–Whitney U and T-Student tests were used. For comparisons between more than two groups, the Kruskal–Wallis ANOVA analysis was utilized, and for post hoc analysis, Tukey’s HSD (honest significant difference) test was employed. The obtained results were presented as the mean, median (middle value), minimum and maximum values, and lower and upper quartile values (respectively, the 25th and 75th percentile). In searching for correlations between examined parameters and to determine the strength of these correlations, Spearman’s nonparametric rank correlation was used. For assessing the significance of differentiation in proportions, the chi-squared independence test was applied (gender). In the verification of all statistical hypotheses, a significance level of α < 0.05 was considered significant.

## 4. Results

### 4.1. BMI, %EBMIL, %EWL, %TWL, Weight Loss

**Figure 1:**
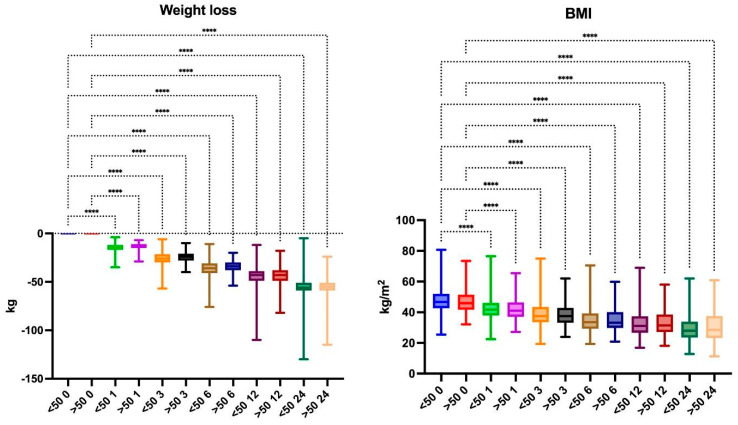
Weight loss and BMI changes at each follow-up. ****—*p* < 0.0001.

The patients were divided into two groups: below (n = 539) and above 50 years old (n = 247). The mean preoperative BMI was 47.71 kg/m^2^ and 47.01 kg/m^2^, respectively. After 24 months of follow-up, mean BMI decreased to 29.03 kg/m^2^ and 30.73 kg/m^2^, respectively, with no significant differences between groups. Mean weight loss was 54.82 kg in the younger group and 54.56 kg in the older group, without significant differences (Figure 1). Patients under 50 years old and patients 50 years old and above showed significant weight loss, with %EBMIL medians at 24 months of 75.22% and 76.44% (Table 2), respectively; the mean %TWL was 30.5% and 30.1% (Table 3) and the mean %EWL was 76.34% and 77.51% with no statistically significant differences between the groups (*p* > 0.05 at all intervals and parameters) (Table 4).

### 4.2. HbA1C and Fasting Glucose Levels

Mean preoperative HbA1C concentrations were significantly different (*p* < 0.0001) at 5.77% (<50 years old) and 6.25% (>50 years old) and became comparable (*p* > 0.9999)—5.66% and 5.53%—after 2 years.

**Figure 2:**
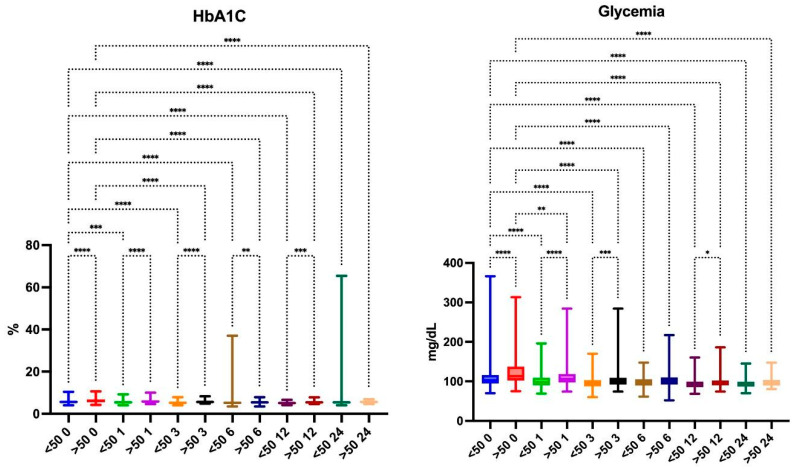
HbA1C and glycemia changes at each follow-up. ****—*p* < 0.0001; ***—0.0001 ≤ *p* ≥ 0.001; **—0.001< *p* ≥ 0.01; *—0.01< *p* > 0.05.

Mean preoperative fasting glucose levels were significantly higher in the group over 50 years old (*p* < 0.0001) throughout 18 months of observation, with no significant differences after two years of (*p* = 0.1162) (Figure 2).

### 4.3. Triglycerides, LDL, HDL, Total Cholesterol Levels

Statistically significant decreases in levels of triglycerides, LDL, HDL and total cholesterol levels were observed in both groups with no significant differences between them. Mean total cholesterol concentrations were 198 mg/dL and 206.4 mg/dL for groups younger and older than 50 years old before the surgery, respectively, and reached 184.9 mg/dL and 193 mg/dL after 24 months. Mean LDL levels were 136.2 mg/dL and 141.0 mg/dL before surgery and fell to 116.7 mg/dL and 121.0 mg/dL after two years. Mean triglycerides levels were 159.4 mg/dL and 172.3 mg/dL and fell to 114.4 mg/dL and 123.6 mg/dL. Mean HDL levels were 50.19 mg/dL and 51.07 mg/dL, reaching 54.65 mg/dL and 54.69 mg/dL. The differences were statistically insignificant when comparing the two groups for all parameters but were significant when comparing the beginning and end of the observations in each group (Table 5).

### 4.4. Hypertension

In the younger group of patients, 68 (12.62%) were diagnosed with hypertension, whereas, in the older group, 60 (24.29%) patients were diagnosed. The mean time since the diagnosis of hypertension was 8.67 years for patients in the under 50 years old group and 13.88 years in the over 50 years old group. The difference was statistically significant *p* = 0.0008. A total of 17 (25%) patients under 50 years old completely discontinued their antihypertensive treatment and 14 (21%) reduced the dosages of medications as compared to 13 (22%) patients in over 50 years old group discontinuing medications and 9 (15%) reducing dosages. Statistical analysis revealed no significant differences in the proportion of patients who discontinued medication (*p* = 0.308) or reduced their medication dosage (*p* = 0.210) between the two age groups.

### 4.5. Type 2 Diabetes Mellitus

In the younger group of patients, 50 (9.28%) were diagnosed with type 2 diabetes mellitus, whereas, in the older group, it was 47 (19.02%) patients. The mean time since diagnosis of T2DM was 8.67 years for patients in the under 50 years old group and 7.59 years in the over 50 years old group. The difference was statistically significant *p* = 0.0017. In total, 4 (8%) patients under 50 years old completely discontinued their antidiabetic treatment and 10 (20%) reduced the dosages of medications as compared to 6 (13%) patients in over 50 years old group discontinuing medications and 8 (17%) reducing dosages. Statistical analysis revealed no significant differences in the proportion of patients who discontinued medication (*p* = 0.662) or reduced their medication dosage (*p* = 0.908) between the two age groups.

## 5. Discussion

The management of metabolic syndrome requires a multifaceted approach that includes lifestyle interventions, pharmacotherapy and surgical procedures [22]. Lifestyle modifications, such as a calorie-restricted diet, regular aerobic and resistance exercise, as well as behavioral therapy, form the cornerstone of treatment, aiming to reduce visceral adiposity, improve insulin sensitivity and ameliorate dyslipidemia. Pharmacotherapy is employed to target the individual components of metabolic syndrome: antihypertensive agents (e.g., ACE inhibitors, ARBs and calcium channel blockers) are used to manage hypertension; statins and fibrates are prescribed to correct dyslipidemia by lowering LDL cholesterol and triglycerides while raising HDL cholesterol; and insulin sensitizers, such as metformin and thiazolidinediones, are used to enhance glucose uptake and reduce insulin resistance. Emerging pharmacotherapies, such as GLP-1 receptor agonists (e.g., liraglutide) and SGLT2 inhibitors (e.g., empagliflozin), offer promising adjunctive treatments by promoting weight loss, enhancing satiety, reducing hepatic glucose production and increasing urinary glucose excretion, thereby addressing multiple facets of metabolic syndrome simultaneously [23]. An integrated treatment strategy that combines these modalities is often necessary to effectively mitigate the risk of cardiovascular disease and type 2 diabetes in patients with metabolic syndrome.

In this study, we investigated the effect of LSG on components of metabolic syndrome in patients over and under 50 years old. Our results indicated no significant differences in the response to treatment between these groups. Both groups of patients were quite similar in terms of preoperative BMI and comorbidities. The data we gathered and analyzed reveal several key points regarding the efficacy of LSG in not only addressing obesity, which is often the primary indication for the surgery, but also in resolving the components of metabolic syndrome.

Our study underscores the significant role of LSG in weight reduction among older patients. Obesity, a major contributor to metabolic syndrome, poses unique challenges in the elderly due to age-related changes in body composition and metabolism [24,25]. The substantial weight loss, observed in our study participants post-LSG, indicates the procedure’s effectiveness, aligning with previous research that has demonstrated LSG’s utility in achieving and maintaining long-term weight loss [26,27,28,29].

The older cohort of patients demonstrated a prolonged history of type 2 diabetes and hypertension compared to younger subjects, but rates of remission were the same. Furthermore, fasting glucose and HbA1C levels were significantly higher in the older population, but we found no significant differences only after two years of observation. Our data support previous findings on bariatric-metabolic surgery being part of treatment of type 2 diabetes [30].

Our findings indicate that there were no significant differences in lipid parameters between the two age groups throughout the study. The LDL, HDL, TG and total cholesterol levels remained consistent across both cohorts, suggesting that age may not significantly influence these aspects of lipid metabolism when both groups are subjected to similar treatment protocols. This uniformity in lipid profiles might reflect the efficacy of the treatments employed, which appeared to be equally effective across different age demographics. It aligns with previous findings by Wysocki et al. [31].

Furthermore, the improvement in metabolic syndrome parameters following LSG in our study group is noteworthy. The normalization of blood pressure, improved lipid profiles, and better glycemic control post-surgery provide compelling evidence of LSG’s role in mitigating the risks associated with metabolic syndrome. These changes are particularly crucial in the over-50 age group, where the risk of cardiovascular events and type 2 diabetes is already elevated.

The main limitation of our study is the relatively short follow-up period, which may not capture persistence and the long-term treatment effects of the operation. Additionally, there is lack of data on waist circumference after surgery and blood pressure measurements after surgery. Further investigation of other factors in treatment responsiveness such as patients compliance, lifestyle, other comorbidities and genetic factors may provide deeper insights into a more personalized treatment approach.

In conclusion, our study adds to the growing body of evidence supporting the use of laparoscopic sleeve gastrectomy as a valuable therapeutic tool in the management of metabolic syndrome in patients over 50 years of age. The positive outcomes observed in terms of weight loss, metabolic improvement and overall safety profile position LSG as a viable option in addressing the multifaceted challenges posed by this syndrome in an aging population.

## 6. Conclusions

Laparoscopic sleeve gastrectomy is an effective method for the treatment of components of metabolic syndrome, regardless of age. While our results are promising, a longer-term follow-up is essential to fully understand the sustainability of these outcomes in the elderly population. Moreover, given the complex interplay of age-related physiological changes, comorbidities and nutritional needs, multidisciplinary management post-LSG is crucial for ensuring optimal outcomes.

## Figures and Tables

**Table 1:** Characteristics of patients.

	<50 Years Old	>50 Years Old	∑
Males	242 (30.8%)	112 (14.2%)	354 (45.2%)
Females	296 (37.7%)	135 (17.2%)	431 (54.8%)
Mean Age	37.6 years	56.9 years	43.7 years
Mean preoperative BMI	47.7 kg/m^2^	47.01 kg/m^2^	47.5 kg/m^2^
Mean preoperative weight	142.8 kg	134.8 kg	140.3 kg
Patients with hypertension	68	60	128
Patients with t.2 diabetes mellitus	50	47	97

<50 years old—patients under 50 years old; >50 years old—patients over 50 years old.

**Table 2:** Median %EBMIL.

Time Interval	Under 50 Years Old %EBMIL Median	Over 50 Years Old %EBMIL Median	*p*-Value
1 month	18.52	19.67	0.82
3 months	34.89	36.23	0.73
6 months	50.15	51.89	0.78
12 months	63.45	64.89	0.76
24 months	75.22	76.44	0.79

**Table 3:** Mean %TWL.

Time Interval	Under 50 Years Old %TWL Mean	Over 50 Years Old %TWL Mean	*p*-Value
1 month	6.50	6.30	0.78
3 months	12.80	12.45	0.73
6 months	18.95	18.60	0.69
12 months	24.20	23.85	0.68
24 months	30.50	30.10	0.65

**Table 4:** Mean %EWL.

Time Interval	Under 50 Years Old %EWL Mean	Over 50 Years Old %EWL Mean	*p*-Value
1 month	22.34	23.12	0.56
3 months	37.45	38.29	0.50
6 months	52.11	53.04	0.47
12 months	65.27	66.45	0.42
24 months	76.34	77.51	0.38

**Table 5:** Changes in tested parameters in time.

	Age Group and Time of Observation in Months											
	<50 0	>50 0	<50 1	>50 1	<50 3	>50 3	<50 6	>50 6	<50 12	>50 12	<50 24	>50 24
HbA1c (%)	5.771 *	6.245 *	5.520 *	5.942 *	5.318 *	5.657 *	5.240 *	5.382 *	5.216 *	5.473 *	5.656	5.525
Fasting glucose (mg/dL)	111.8 *	128.5 *	101.0 *	111.1 *	96.83 *	104.3 *	97.69	102.7	92.77 *	99.74 *	93.48	98.31
HDL (mg/dL)	50.19	51.07	37.12	39.36	43.96 *	45.32 *	49.73	52.89	55.76	57.88	54.65	54.69
LDL (mg/dL)	136.2	141.0	115.0	122.6	117.6	125.1	127.3	129.2	116.4	126.9	116.7	121.0
TG (mg/dL)	159.4	172.3	137.5	152.6	121.7	134.5	117.0	113.3	95.72	115.7	114.4	123.6
Total cholesterol (mg/dL)	198.3	206.4	171.8	182.5	175.7	186.3	191.0	198.5	179.0	193.3	184.9	193.0
Weight loss (kg)	0.000	0.000	−14.83	−13.80	−26.59	−24.76	−37.06	−34.40	−44.55	−43.22	−54.82	−54.56
BMI (kg/m^2^)	47.71	47.01	42.40	42.22	38.73	38.69	34.79	35.23	32.31	33.09	29.03	30.73

*—*p* < 0.05 comparing to the second group at the same time interval: <50—group of patients under 50 years old; >50—group of patients over 50 years old; 0—before surgery; 1, 3, 6, 12, 24—months after surgery.

## Data Availability

The original contributions presented in the study are included in the article, further inquiries can be directed to the corresponding author.
